# Wound Infections in Adult Patients after Berlin Heart^®^ EXCOR Biventricular Assist Device Implantation

**DOI:** 10.3390/life12101550

**Published:** 2022-10-06

**Authors:** Jamila Kremer, Abbas El-Dor, Rasmus Rivinius, Philipp Schlegel, Wiebke Sommer, Gregor Warnecke, Matthias Karck, Arjang Ruhparwar, Anna L. Meyer

**Affiliations:** 1 Department of Cardiothoracic Surgery, University Hospital Heidelberg, 69120 Heidelberg, Germany; 2 Department of Cardiology, Angiology and Pneumology, Heidelberg University Hospital, 69120 Heidelberg, Germany; 3 Clinic of Thoracic and Cardiovascular Surgery, Essen University Hospital, 45147 Essen, Germany

**Keywords:** Berlin Heart^®^ EXCOR system, mechanical circulatory support, heart transplantation, infections, biventricular assist device (BiVAD)

## Abstract

**Simple Summary:**

Our study shows that the Berlin Heart^®^ EXCOR as a biventricular assist device in adult patients is an effective strategy in heart failure medicine. Our results may increase the use of the EXCOR with acceptable clinical outcomes despite long support times. With ongoing research to implement the EXCOR as a permanent right ventricular assist device, our results highlight possible arising complications.

**Abstract:**

The Berlin Heart^®^ EXCOR is a paracorporeal, pulsatile ventricular assist device used in patients of all age groups. However, adolescent and adult patients on EXCOR support are scarcely explored. Herein, we present a detailed description of infectious complications in this patient cohort. From 2006 to 2020, 58 patients received a biventricular assist device (BiVAD) at our institution and were included in this study. Postoperative infections were assessed after BiVAD implantation and subsequent heart transplantation (HTx). A Berlin Heart^®^ EXCOR BiVAD was implanted as a bridge to transplantation in 58 patients (12–64 years). Most patients were INTERMACS I, and their median age was 49 years. Wound infections (WI) specific to the ventricular assist device (VAD) occurred in 31 (53.4%) patients with a mean time of 113 ± 155 days after BiVAD implantation. HTx was performed in 30 (51.7%) patients and thereof 10 (33.3%) patients developed at least one WI post-HTx. The mean time of WI after HTx was 17 ± 14 days. In four cases, WIs were caused by the same pathogen as before HTx. According to our institutional BiVAD wound classification, the mean wound score was 3. The VAD-specific wound infections were manageable and did not increase mortality nor precluded HTx in Berlin Heart^®^ EXCOR patients. No specific risk factors for VAD-specific wound infections could be identified.

## 1. Introduction

Ventricular assist devices (VAD) have been used as bridge to recovery (BTR), bridge to transplantation (BTT), and, most recently, as destination therapy in heart failure patients [1,2]. 

The Berlin Heart**^®^** EXCOR is a paracorporeal, pulsatile, and pneumatically-driven VAD used in patients of all age groups for short-, mid-, and long-term cardiac support. It can provide durable support to the left ventricle (LV), right ventricle (RV), or both ventricles and can be used as BTR or BTT. It is the only system worldwide that is officially designed and licensed for pediatric patients [3,4,5]. Pump chambers are available in six different sizes.

Starting with pulsatile systems, further VAD types, such as continuous flow (CF) devices, have evolved. However, CF-VADs are not suitable for all patients in need of BTT therapy [6]. Adult patients with complex congenital heart diseases or biventricular heart failure (HF) require additional RV support [7]. A recent study by McGiffin et al. reports low operative mortality in patients with two HeartMate 3 (Abbott Laboratories, Chicago, IL, USA) CF-VADs for biventricular support with a 90%-survival after 18 months. In the future, these results need to be confirmed by other multi-center studies [8].

The main complications under VAD support include bleeding, thrombosis, and infections [9]. Infection rates in previous VAD studies vary from 18% to 59% [10,11,12]. Non-VAD-related infections, including pneumonia, bloodstream infections, urinary tract infections, and gastrointestinal infections, have been the leading category of infection in mechanical circulatory support (MCS) patients, followed by VAD-specific infections, mainly including driveline infections (80%) [13,14]. Despite being one of the most common complications in VAD therapy, infections, especially for adult patients, and biventricular assist device (BiVAD) implantations, have, to date, been scarcely studied. 

The aim of this study is to report the rate and characteristics of infections diagnosed in adolescent and adult patients with a Berlin Heart**^®^** EXCOR BiVAD at our institution and to compare those results with the current literature on biventricular MCS in adult patients.

## 2. Materials and Methods

This retrospective single-center study includes all adolescents and adult patients (12–64 years) who received a Berlin Heart**^®^** EXCOR BiVAD from 2006 to 2020 at the University Hospital Heidelberg. The study complies with the Declaration of Helsinki and ethical approval was granted by the ethical committee of the University of Heidelberg, S-601/2020. Patients’ data were collected in a prospective manner until death after BiVAD implantation, successful heart transplantation (HTx), or death after HTx. Postoperative infections were assessed after BiVAD implantation and subsequent HTx for those receiving it.

### 2.1. Definitions

We classified infections according to the definitions of the International Society of Heart and Lung Transplantation (ISHLT) [15]. These definitions have been divided into three sections: VAD-specific infections (VAD-S), VAD-related infections (VAD-R) and non-VAD infections (N-VAD). 

The definitions of the Centers for Disease Control and Prevention (CDC) were additionally used to allow a more accurate distinguishment between superficial and deep WI [16]. Superficial WIs are limited to the skin and subcutaneous tissue, while deep WIs involve deep soft tissue (e.g., fascial and muscle layers). 

**Figure 1:**
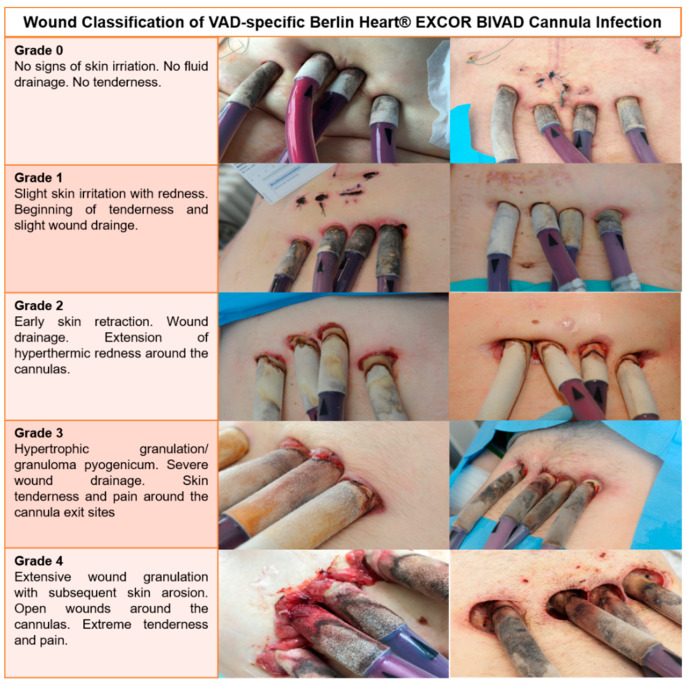
The Heidelberg Berlin Heart^®^ EXCOR cannula wound classification.

We also provide our institution’s VAD wound classification for cannula and drive-line infections (Figure 1). Wounds are categorized according to clinical appearance.

Bloodstream infections were defined as cases in which one or more pathogens were isolated from one or more blood cultures. Pathogens of doubtful significance (e.g., coagulase-negative Staphylococcus species) were counted when present in at least two blood cultures with clinical manifestation. Furthermore, primary BSIs were distinguished from central venous catheter (CVC)-related BSIs. According to a recommendation from the ISHLT Guidelines BSIs were considered CVC-related if differential time to positivity was at least two hours between catheter blood and peripheral blood collection, or if the same pathogen was recovered from blood and catheter-tip cultures [15].

### 2.2. Statistics

Data are presented as means and standard deviations (SD), medians and interquartile ranges (IQR), or numbers and percentages. Statistical comparisons between patients with and without VAD-S WI were performed with an unpaired t-test for normally distributed data, Mann–Whitney U-test for nonparametric data and Fisher exact test for categorical data. Kaplan–Meier curves were generated using “VAD-S wound infections” as the event. For identifying pre-implant and implant-related risk factors associated with developing VAD-S WI a multivariable logistic regression analysis was performed. Statistical analyses were performed with version 25 of the SPSS software package.

## 3. Results

### 3.1. Patient Characteristics

A total of 58 patients received a Berlin Heart^®^ EXCOR BiVAD from 2006 to 2020. The treatment intention was BTT for all patients. Operative mortality within the first 30 days after BiVAD implantation was 25.9%. At least one VAD-S WI occurred in 31 (53.4%) patients during BiVAD support (“with WI”), while 27 (46.6%) patients remained free from wound infections (“without WI”). The demographic data are shown in Table 1. The median age was 49 years (IQR = 34–55 years) and 82.8% were male patients. Women tended to be younger (41 vs. 51, *p* = 0.085). Five (8.6%) adolescent patients older than 12 years were included in this analysis. Most patients were INTERMACS I. Leading heart failure etiology that necessitated BiVAD therapy was non-ischemic cardiomyopathy (*n* = 36), followed by ischemic cardiomyopathy (*n* = 13) and myocarditis (*n* = 9). 

Intraoperative data from BiVAD implantations are listed in Table 2. Four patients received concomitant cardiac surgery to the BiVAD implantation, including mitral valve replacements (*n* = 2), aortic valve replacement (*n* = 1), and patent foramen ovale closure (*n* = 1).

### 3.2. Overall Clinical Outcome after BiVAD Implantation

Overall mortality was 48.3% with significantly higher mortality rates for patients without WI, 18 (66.7%) vs. 10 (32.3%), *p* = 0.010. Causes of death were multi-organ failure (*n* = 7), septic shock (*n* = 5), cardiogenic shock (*n* = 4), bleeding (*n* = 4), cerebral bleeding (*n* = 4), and embolic event (*n* = 3). 

Patients with WI died within a mean time of 123 ± 154 days, while patients without WI had a mean time to death of 35 ± 51 days after BiVAD implantation (***p* = 0.006**). Similarly, patients with WI were transplanted on average after 390 ± 249 days, while patients without WI had a mean time to HTx of 142 ± 70 days (***p* = 0.007**). However, patients with early WI (within 30 days of BiVAD implantation) had a mean time to HTx of 189 ± 149 days. Patients who received a BiVAD before 2015 were transplanted faster than patients who were bridged to HTx thereafter. However, this time difference was not statistically different (169 ± 227 vs. 232 ± 220 days, *p* = 0.252).

### 3.3. Infections Post-BiVAD

All patients were assessed for infections after BiVAD implantation considering the CDC classification and ISHLT criteria. 

The mean BiVAD support time without WI was 91 ± 125 days. Univariate and multivariate analyses were also performed to identify possible predictors. More recent BiVAD implantations (since 2015) (HR 2.413; 95% CI 1.092–5.334) and the application of an ePTFE Goretex membrane (HR 2.726; 95% CI 1.087–6.833) were independent risk factors for the complication “wound infection”, as shown in Table 3.

#### 3.3.1. ISHLT Criteria 

**Figure 2:**
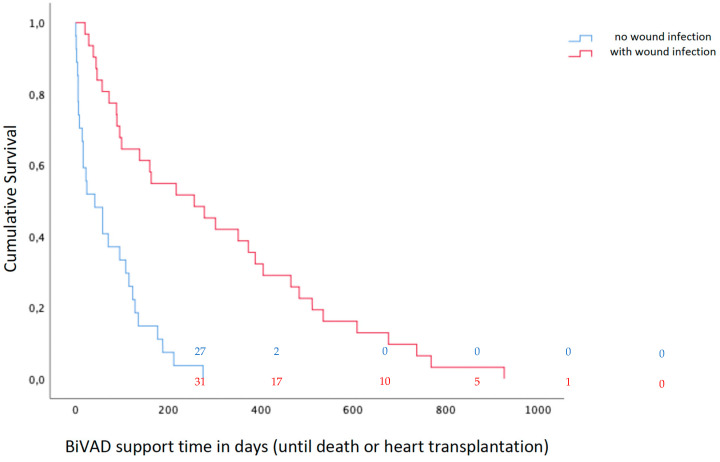
Kaplan–Meier survival curve of patients with versus those without VAD-S wound infections. Patients at risk are shown in coloured numbers every 200 days for patients with wound infection versus those without wound infection.

VAD-specific infections occurred in 31 patients, all being cannula/wound infections. VAD-S WI per patient-year was 1.00. The mean time to VAD-S WI was 113 ± 158 days. BiVAD support time of patients with VAD-S WI averaged 304 ± 254 days, while patients without WI had a mean BiVAD support time of 71 ± 76 days (***p* < 0.001)**. Figure 2 compares the BiVAD support times of patients with versus those without VAD-S WI. Eight patients had an additional VAD-related infection. Of the 11 VAD-related infections, 10 were primary or catheter-related bloodstream infections (BSI) and 1 was mediastinitis. At least one non-VAD-related infection was documented in 30 patients, including 21 urinary tract infections, 7 infections with Clostridium difficile, and 8 lower respiratory tract infections.

**Figure 3:**
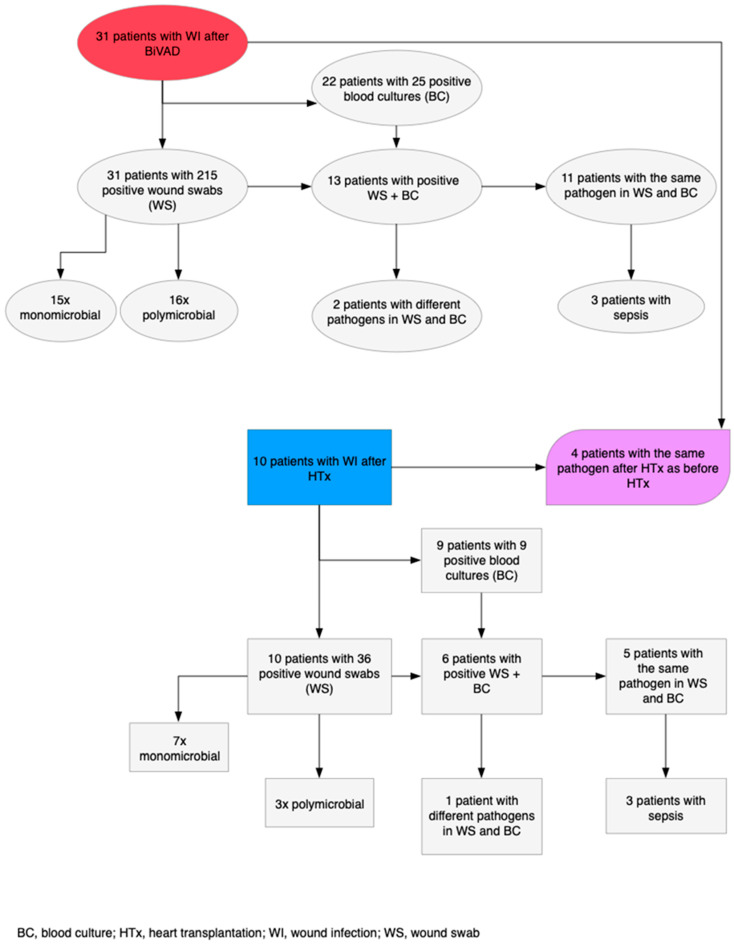
Flowchart of microbiological examination results of wound swabs from infection sites.

All 31 patients with VAD-S WI had at least one positive wound swab with 16 polymicrobial vs. 15 monomicrobial etiologies. Enterococcus and Candida species predominated with 12 cases each. In addition to positive wound swabs, 13 patients had positive blood cultures with 11 blood cultures containing identical pathogens to the wound swabs (Figure 3). The most frequent pathogens occurring in both examinations were coagulase-negative Staphylococcus (*n* = 8) and Pseudomonas aeruginosa (*n* = 2). Out of these 13 patients, 3 developed a pathogen-related sepsis.

The pathogens causing VAD-related or non-VAD-related infections are listed in Table 4. For VAD-related infections, coagulase-negative Staphylococcus and Candida species were the main species. For non-VAD-related infections the most common etiologies were gram-negative bacteria (mainly *Escherichia coli*), mainly causing urinary tract infections, followed by clostridium difficile infections. 

#### 3.3.2. CDC Classification

In total, 31 (53.4%) patients matched the criteria for a wound infection with superficial WIs in 13 patients, equal to 0.42 WIs per patient-year, while 18 developed deeper local WIs, equaling 0.58 WIs per patient-year. Although superficial WIs had significantly more monomicrobial etiologies (*p* = 0.052), deep WIs were caused more frequently by polymicrobial floras (*p* = 0.052) (Table 5). Enterococcus species were more often found in deep WIs (*p* = 0.029). Pseudomonas aeruginosa was also found in deeper WIs (*p* = 0.073). In total, 28 of 31 patients with WI were treated with calculated antibiotic therapy and additional surgical treatment with vacuum-assisted therapy. The three remaining patients had superficial wound healing disorders, which could be treated without antibiotics. 

In regards to our institutional BiVAD cannula infection classification: In 31 patients with VAD-specific infection, the wound conditions were photo-documented or specified in writing. Figure 1 shows wound examples of our patients with corresponding clinical descriptions and classifications. The mean wound score for all 31 patients was 3. In the group who died on BiVAD support, the mean wound score was 2 compared to a wound score of 3 in the group who was bridged to HTx, outside the level of statistical significance.

### 3.4. Wound Infections Post-HTx

During their clinical course, 30 patients received a HTx and 10 (33.3%) developed at least one WI afterwards. Operative mortality within 30 days of HTx was 3.3%. Follow-up time made up a total of 51.662 days resulting in a WI rate of 0.07 WIs per patient year after HTx. There were eight superficial WIs and two deep WIs occurring with a mean time of 17 ± 14 days. The time range of WIs after HTx went from 0 to 40 days. In four cases (12.9%), WIs were caused by the same pathogen as before HTx. All these recurring WIs happened in the first 17 days after HTx. The respective pathogens were in two cases Pseudomonas aeruginosa, one case coagulase-negative Staphylococcus and one case Vancomycin-resistant Enterococcus. The microbiological examination results after HTx are illustrated in Figure 3. 

The 10 patients with WIs after HTx had 36 positive wound swabs and 9 positive blood cultures with 6 patients being positive in both. The pathogens common to both examinations were Enterococcus species (*n* = 3) and Pseudomonas aeruginosa (*n* = 2). Of these patients, three developed a pathogen-related sepsis. Of the patients who had the same pathogen as before HTx, two were positive in both the wound swab and blood culture. Antibiotic therapy was needed in 8 (80%) of the 10 WI patients.

During the follow-up time, 11 patients died after receiving HTx with a 30-day-survival of 96.7%, one-year-survival of 76.7% and three-year-survival of 69.7%. 

Causes of death after HTx were septic shock (*n* = 4), cardiogenic shock (*n* = 3), hemorrhagic stroke (*n* = 1) and three causes of death are undetermined due to loss to follow-up. Only one death due to septic shock was attributable to the WI. Perforated diverticulitis of the sigmoid colon, CVC-related BSIs and pancreatitis were the causes of septic shock leading to death in the other patients.

The risk factor analysis could rule out WI as a risk factor for mortality after HTx (*p* = 0.292).

## 4. Discussion

Many countries, including Germany, struggle with a shortage of organ donors, while the number of patients in need of HTx due to acute or chronic heart failure is increasing. Therefore, MCS is suitable for long support times as BTT becomes more important in the clinical routine [1,5,17]. Occurring in increasing numbers in VAD-supported patients, related complications present a major problem in aftercare.

Data regarding the usage of Berlin Heart**^®^** EXCOR devices, especially in a biventricular configuration, is limited [5]. Although VAD infections have been the focus of several pediatric studies, their impact on adolescent and adult patients, who carry different etiologies, comorbidities, and characteristics, is still not well documented. 

Two larger German studies with 122 and 29 Berlin Heart**^®^** EXCOR patients, respectively, including only pediatric patients (median ages: 3 and 9 years), described cannula infection rates of 67% and 13.8%, respectively [18,19]. In other recent studies of adult Berlin Heart**^®^** EXCOR patients, VAD-S infections occurred in 14–33.3% after implantation. However, not all devices were used in a biventricular configuration [7,11,20]. Herein, VAD-specific (cannula) WI appeared at a higher rate than in other studies (53.4%). However, our patient cohort had longer support times (195 days vs. 135/92 days). This time difference can be explained by the short transplantation waiting lists and high donation rates in other countries, while a shortage of organ donors remains one of the main concerns in the transplant community in Germany. Severe wound infections are not an early complication of VAD therapy and the incidence rises with the length of support [21]. None of the patients who died within the first month after implantation developed a severe WI. Hence, the risk for infection is inevitably higher for our patients on the transplant waiting list.

More recent BiVAD implantations (after 2015) and the application of a Goretex membrane were independent risk factors for the complication “wound infection”. Although the Goretex membrane may prevent adhesions after BiVAD implantation, it also increases the risk of infections. However, it is important to mention that the higher VAD-S WI rates in recent BiVAD patients indicate a better clinical outcome on prolonged BiVAD support and better survival on MCS.

Our results show that VAD-S infections (53.4%) were the most common, followed by N-VAD infections (50.8%) and VAD-R infections (19.0%). This stands in contrast to other studies, in which N-VAD infections were the largest category [11,13,20]. BiVAD support times in our study were very long and the number of VAD-S infections consequently rose. This underlines the difficulties of VAD support in countries with long waiting lists for HTx. Most importantly, all our patients received Berlin Heart**^®^** EXCOR devices in a biventricular configuration, representing a severely ill patient group with most patients in INTERMACS I. These patients were all in dire need of a sufficient failure HF treatment strategy. The time to HTx for patients surviving the first month, without the development of WI, was approximately five months. Patients with VAD-S WI were transplanted on average after 13 months. This fact does not lead to the conclusion that HTx was delayed due to WI, but rather it indicates that an extended waiting time will inevitably lead to severer VAD-S or VAD-R infections during the time of BiVAD treatment. Overall, 34 clinical outcomes were complicated by VAD-S (*n* = 31) and/or VAD-R (*n* = 11) infections after BiVAD implantation. All 31 VAD-S infections were cannula/wound infections occurring within a mean time of over 113 days. 

So far, only one study has assessed the potential relapse of VAD-associated infection after HTx [11]. In four of our cases (12.9%), WIs were caused by the same pathogen as before HTx. As with cases described in the literature, all these recurring WIs happened in the first 30 days after HTx. This suggests that VAD-S infections after implantation increase the risk of further infection by the same pathogen after HTx. Therefore, it is necessary to address the importance of post-surgical antibiotic therapy to prevent such infection relapses.

Wound infection was not a risk factor for mortality after BiVAD implantation and after HTx. This result is comparable to other studies on infections in mechanical circulatory support patients [22,23]. Here we demonstrate that the Berlin Heart**^®^** EXCOR devices in a biventricular configuration are an effective strategy for patients in need of long-term MCS. With the prospect of ongoing research to implement the Berlin Heart**^®^** EXCOR device as a permanent RV support, these results point out possible arising complications.

The limitations of this study include the small patient cohort, as well as its retrospective nature, which limited further statistical analyses and its descriptive character.

## 5. Conclusions

The study may lead to more frequent use of the Berlin Heart**^®^** EXCOR in severely ill patients with biventricular heart failure, considering the acceptable clinical outcome despite long VAD support times. VAD-specific infections remain a major complication for Berlin Heart**^®^** EXCOR patients; however, they are manageable and did not increase mortality nor preclude HTx. Further improvements in multidisciplinary management and prophylactic measures are needed to reduce infection rates after VAD implantation and after HTx.

## Figures and Tables

**Table 1:** Patient characteristics of the study population at the time of BiVAD implantation based on post-implant wound infection.

Characteristics	With Wound Infection (*n* = 31)	Without Wound Infection (*n* = 27)	*p*-Value
**Age** years	47 (34–55)	49 (34–53)	0.668
**Male** sex	28 (90.3%)	20 (74.1%)	0.105
**Body mass index**, kg/m^2^	26.9 (21.3–30.9)	27.0 (22.4–33.2)	0.725
**Body surface area**, m^2^	2.02 (1.85–2.2)	1.97 (1.77–2.08)	0.171
**Time of BiVAD**			**0.027**
2006–2014	14 (45.2%)	20 (74.1%)	
2015–2020	17 (54.8%)	7 (25.9%)	
**INTERMACS Profile**			0.585
1	20 (64.5%)	20 (74.1%)	
2	8 (25.8%)	3 (11.1%)	
3	3 (9.7%)	4 (14.8%)	
**CMP aetiology**			0.928
Non-ischemic	21 (67.7%)	15 (55.6%)	
Ischemic	6 (19.4%)	7 (25.9%)	
Myocarditis	4 (12.9%)	5 (18.5%)	
**Comorbidities**			
Dialysis pre-BiVAD	9 (29.0%)	9 (33.3%)	0.726
Diabetes mellitus	10 (32.3%)	7 (25.9%)	0.668
Hyperlipidaemia	8 (25.8)	7 (25.9%)	0.992
Arterial hypertension	12 (38.7%)	11 (40.7%)	0.876
Pulmonary hypertension	17 (54.8%)	14 (51.9%)	0.242
**Previous cardiac surgery**	4 (12.9%)	7 (25.9%)	0.211
**Pre-operative support**			
IABP	7 (22.6%)	8 (29.6%)	0.544
ECLS	14 (45.2%)	15 (55.6%)	0.434
**Urgency of BiVAD**			0.387
Elective	4 (12.9%)	2 (7.4%)	
Urgent	16 (51.6%)	12 (44.4%)	
Emergency	7 (22.6%)	10 (37.0%)	
Ultima ratio/reanimation	4 (12.9%)	3 (11.1%)	

Continuous data are presented as median (IQR) and categoric data as number (%). BiVAD, biventricular assist device; INTERMACS, Interagency Registry for Mechanically Assisted Circulatory Support; CMP, cardiomyopathy; IABP, intra-aortic balloon pump; ECLS, extracorporeal life support.

**Table 2:** Intraoperative data from BiVAD implantation based on post-implant wound infection.

	With Wound Infection (*n* = 31)	Without Wound Infection (*n* = 27)	*p*-Value
**Cardiopulmonary bypass time** minutes	217.70 (138–336)	221.23 (98–494)	0.532
**Aortic cross-clamp time** minutes	33.97 (0–291)	27.23 (0–215)	0.869
**Concomitant cardiac surgery after BiVAD implantation**	2 (6.5%)	2 (7.4%)	0.887
**Gortex membrane**	23 (74.2%)	15 (55.6%)	0.140
**Minimal body temperature** °C	35.23 (32–37)	34.41 (28–36.8)	0.084
**Packed red blood cells** units	9.29 ± 6.43	10.93 ± 7.85	0.462
**Fresh frozen plasma** units	5.24 ± 6.11	5.47 ± 5.93	0.842
**Surgery time** minutes	421.32 (273–640)	411.00 (145–980)	0.322

Continuous data are presented as mean (range) and categoric data as number (%).

**Table 3:** Final Cox model on independent risk factors for the complication “wound infection”.

	B	SE	Wald	df	Sig.	Exp(B)	95.0% CI for Exp(B)	
							Lower	Upper
Time of BiVAD	0.881	0.405	4.741	1	0.029	2.413	1.092	5.334
Gortex membrane	1.003	0.469	4.572	1	0.033	2.726	1.087	6.833

**Table 4:** Microbiological results comparing superficial with deep WI.

	Superficial Wound Infection (*n* = 13)	Deep Wound Infection (*n* = 18)	*p*-Value
**Polymicrobial**	4	12	0.052
**Monomicrobial**	9	6	0.052
**Gram-positive**	5	13	0.064
S. aureus	4	5	0.859
Enterococcus spec.	1	8	**0.029**
**Gram-negative**	2	10	**0.026**
P. aeruginosa	0	4	0.073
**Candida spec.**	4	5	0.859
Others *	7	15	0.079

S. aureus = Staphylococcus aureus; Spec. = species; gram-negative = gram-negative bacteria such as E. coli, Klebsiella pneumoniae; P. aeruginosa = Pseudomonas aeruginosa. Others * = Corynebacterium, Acinetobacter baumanii, Actinomyces species, Stenotrophomonas maltophilia, coagulase-negative Staphylococcus.

**Table 5:** Microbiological aetiologies of VAD-R and N-VAD infections after BiVAD implantation.

Pathogen (VAD-R)	Number of Patients	Pathogen (N-VAD)	Number of Patients
**Gram-positive**	5	**Gram-positive**	4
Enterococcus spec.	1	Enterococcus spec.	4
Coagulase-negative Staphylococcus	4		
**Gram-negative**	4	**Gram-negative**	14
P. aeruginosa	3	P. aeruginosa	4
**Candida spec.**	4	**Candida spec.**	6
		**Clostridium difficile**	7
		**Herpes simplex virus**	1

**VAD-R = VAD-related; N-VAD = non-VAD-related; S.** aureus = Staphylococcus aureus; Spec. = species; gram-negative = gram-negative bacteria such as E. coli and Klebsiella pneumoniae; P. aeruginosa = Pseudomonas aeruginosa.

## Data Availability

Not applicable.
